# Assessment of 2021/22 influenza epidemic scenarios in Italy during SARS-CoV-2 outbreak

**DOI:** 10.1371/journal.pone.0282782

**Published:** 2023-03-09

**Authors:** Maria Chironna, Giovanni Dipierro, Jean Marie Franzini, Giancarlo Icardi, Daniela Loconsole, Elena Pariani, Stefano Pastore, Marco Volpe

**Affiliations:** 1 Department of interdisciplinary Medicine, University of Bari, Aldo Moro Policlinico, Bari, Italy; 2 G-nous s.r.l., Bari, Italy; 3 BIP Life Sciences, BIP spa, Milan, Italy; 4 Department of Health’s Science (DiSSal), University of Genoa, Genoa, Italy; 5 Hygiene Unit, San Martino Policlinico Hospital-IRCCS for Oncology and Neurosciences, Genoa, Italy; 6 Department of Biomedical Sciences for Health, University of Milan, Milan, Italy; Fiji National University, FIJI

## Abstract

Global mitigation strategies to tackle the threat posed by SARS-CoV-2 have produced a significant decrease of the severity of 2020/21 seasonal influenza, which might result in a reduced population natural immunity for the upcoming 2021/22 influenza season. To predict the spread of influenza virus in Italy and the impact of prevention and control measures, we present an age-structured Susceptible-Exposed-Infectious-Removed (SEIR) model including the role of social mixing patterns and the impact of age-stratified vaccination strategies and Non-Pharmaceutical Interventions (NPIs) such as school closures, partial lockdown, as well as the adoption of personal protective equipment and the practice of hand hygiene. We find that vaccination campaigns with standard coverage would produce a remarkable mitigation of the spread of the disease in moderate influenza seasons, making the adoption of NPIs unnecessary. However, in case of severe seasonal epidemics, a standard vaccination coverage would not be sufficiently effective in fighting the epidemic, thus implying that a combination with the adoption of NPIs is necessary to contain the disease. Alternatively, our results show that the enhancement of the vaccination coverage would reduce the need to adopt NPIs, thus limiting the economic and social impacts that NPIs might produce. Our results highlight the need to respond to the influenza epidemic by strengthening the vaccination coverage.

## 1. Introduction

The worldwide fight against the threat posed by the Severe Acute Respiratory Syndrome-Coronavirus-2 disease (SARS-CoV-2, mostly known as COVID-19 [1]) has led to a wide implementation of Non-Pharmaceutical Interventions (NPIs) such as partial lockdown, social distancing (e.g., avoiding mass gatherings, closing public entertainment venues and schools), as well as the adoption of Personal Protective Equipment (PPE) and the practice of frequent hand hygiene. These interventions will continue to play a crucial role in public health safety as new COVID-19 variants arise in the next future.

The adoption of these measures since the first months of 2020 have produced a remarkable decrease of the severity of seasonal influenza-like illnesses (ILI) during the 2020/21 season [2–4], with numbers detected being similar to those reported during inter-seasonal periods. Similarly to COVID-19, seasonal influenza (flu) is a highly infectious respiratory disease transmitted primarily by air (drops and aerosols) that can cause serious health complications. In a typical year, the global ILI incidence ranges between 5 and 10 percent for adults and between 20 and 30 percent for children, out of which an estimated 3 to 5 million are severe, leading to about 250,000 to 500,000 flu-related deaths [5]. Although the COVID-19 control measures helped to prevent flu from circulating during the 2020/21 season [6], the decrease of the severity of ILI might imply a lower population immunity for the season 2021/22, since most people have not been exposed to ILI for over a year [7, 8]. This would leave the most vulnerable people at risk of serious illness.

In the light of this, influenza vaccination can cover a key role in limiting the diffusion of seasonal influenza infection, as well as in avoiding the overwhelming of healthcare systems that are already under pressure [9, 10]. Recent studies have shown that the COVID-19 outbreak have produced an increase of adherence to flu vaccination, reaching the highest coverage in the vaccination campaign 2020/21 [11–13]. Moreover, a large number of studies have demonstrated that NPIs have significant effects on the spread of airborne epidemics [14–16]. Due to the continued impact of the COVID-19 pandemic and the unpredictability of the influenza virus, it is therefore timely to investigate the expected flu epidemic scenarios for the upcoming 2021/22 season by taking into account the impact of the currently adopted NPIs and the influenza vaccination strategies in order to support both individual decision-making and policy-making in the fight against flu epidemics. By providing authorities with predictions about the distribution of the ILI over the different age classes, it will be allowed to develop preparedness plans in order to timely respond to a possible pandemic outbreak [17, 18].

As suggested by recent studies (e.g., [19, 20]), the development of mathematical approaches to model the dynamic of the epidemic and the impact of control and prevention measures can provide a tool to assess and compare the effectiveness of different public health measures to control the disease and can be considered a necessary practice to infer valuable information for public health policymakers [21]. Although there are some limitations in modelling real-life situations, mathematical models have been used to characterize the dynamic of previous diseases such as the Ebola virus [22], influenza [23, 24] and COVID-19 [25–28].

This paper aims to explore a wide range of influenza epidemic scenarios for the season 2021/22 in Italy by taking into account the impact of single and multiple control and prevention measures in the spread of the disease for different seasonal severity. The characteristics of the epidemic scenarios are assessed through an age-structured deterministic Susceptible-Exposed-Infectious-Recovered (SEIR) epidemiological model, suitably extended in order to properly model age-structured social mixing and the impact of the control and prevention measures. The investigation of these scenarios is crucial for policymakers to infer the impact of implementing measures and to assess whether vaccination campaign strategies need to be revised to successfully lessen the public health burden.

The paper is structured as follows: Section 2 describes the SEIR epidemiological model and the explored scenarios. Results are presented in Section 3, followed by discussions in Section 4. Finally, the concluding remarks are drawn in Section 5.

## 2. Materials and methods

### 2.1. Epidemic model

We develop an age-structured deterministic compartment model based on the well-known SEIR model [29, 30], originally introduced as an epidemic model for the description of the dynamics of an infectious disease in a homogeneous and well-mixed population. Among the different approaches developed in the past (e.g., [31, 32]), mathematical modelling based on compartmental dynamical systems can often provide essential information of the epidemic dynamics, especially in cases where there is large uncertainty in the impact of the prevention and control measures for containing the spread of the disease. We extend the classical SEIR model through the introduction of age-structuring and the incorporation of the social mixing patterns (e.g., [33, 34]), thus relaxing the assumption of homogeneous mixing across population age groups.

The social mixing of age-structured population in our model is captured through the use of a realistic model of mixing patterns computed from routinely collected socio-demographic data of the Italian population [33] (see Sect. 2.2). We stratify the population into four classes: infants (0–4 years), children (5–14 years), adults (15–64 years) and the elderly (≥ 65 years). The total population is distributed among the different age classes according to the Italian population Census updated to January 1, 2019 [35].

Our model includes the impact of movement restrictions on population mixing by scaling age-structured contact matrices accordingly (see Sect. 2.2). Moreover, the model discriminates between different severity of illness for infected individuals by taking into account moderate cases, defined as infected individuals that require a treatment by General Practitioners (GP) or in Emergency Rooms (ER) and life-threatening cases that require Intensive Care Unit (ICU) treatment. We investigate the characteristics of seasonal influenza without distinguishing between the specific subtypes of influenza virus. However, as described below, the parameters of the model are computed by averaging epidemiological characteristics of two influenza virus subtypes, i.e., H1N1 and H3N2, which are the main subtypes causing seasonal influenza [36].

We assume a population made of individuals subdivided into the following mutually exclusive seven compartments for each age class *i* = 1,…,4,:

1. Susceptible (*S*_*i*_): individuals able to contract the flu,
2. Exposed (*E*_*i*_): susceptible which have been exposed to contagious (exposed and infected) individuals and are able to infect,
3. Infected (*I*_*i*_): infected individuals,
4. GP/ER treated (*G*_*i*_): infected individuals being treated by general practitioners or in emergency rooms,
5. ICU treated (*T*_*i*_): infected individuals requiring critical medical care in ICU,
6. Recovered (*R*_*i*_): individuals removed from the epidemic dynamics by recovering or by getting the vaccination,
7. Deceased (*D*_*i*_): individuals passing away due to the flu.

We consider closed age classes with no births and natural deaths, such that the population number *N*_*i*_ of age class *i* is the sum of individuals in all the compartments of that age class over time *t* (i.e., $N_{i}=S_{i}(t)+E_{i}(t)+I_{i}(t)+G_{i}(t)+T_{i}(t)+R_{i}(t)+D_{i}(t)$). We assume that the probability of reinfection of recovered individuals is negligible, ensuring a lifelong immunity to the influenza upon recovery. The transitions of individuals between different compartments are modelled by a system of seven ordinary differential equations, describing the evolution of the population for each age class *i* = 1,…,4 over time *t*, i.e.,

$$\frac{dS_{i}}{dt}=-\beta_{i}p_{i}S_{i}\sum_{j=1}^{4}M_{ij}\frac{I_{j}+E_{j}}{N_{j}}-\epsilon_{i}(t)c_{i}S_{i},$$ (1)

$$\frac{dE_{i}}{dt}=\beta_{i}p_{i}S_{i}\sum_{j=1}^{4}M_{ij}\frac{I_{j}+E_{j}}{N_{j}}-\sigma E_{i},$$ (2)

$$\frac{dI_{i}}{dt}=\sigma E_{i}-\gamma I_{i}-\mu_{i}I_{i},$$ (3)

$$\frac{dG_{i}}{dt}=a_{i}\gamma I_{i}-\gamma G_{i}-\mu_{i}G_{i},$$ (4)

$$\frac{dT_{i}}{dt}=b_{i}\gamma I_{i}-\gamma T_{i}-\mu_{i}T_{i},$$ (5)

$$\frac{dR_{i}}{dt}=(1-a_{i}-b_{i})\gamma I_{i}+\gamma (G_{i}+T_{i})+\epsilon_{i}(t)c_{i}S_{i},$$ (6)

$$\frac{dD_{i}}{dt}=\mu_{i}(I_{i}+G_{i}+T_{i}),$$ (7)

where the upper-case letters denotes the state variables (*S*_*i*_, *E*_*i*_, *I*_*i*_, *G*_*i*_, *T*_*i*_, *R*_*i*_, *D*_*i*_) introduced above and *M*_*ij*_ indicates the contact matrix (see Sect. 2.2), defined as average number of daily contacts between an individual in age class *i* with an individual in age class *j*, where *i*, *j* = 1,…,4. The lower-case letters denote the transition rates and the fraction of individual moving between different compartments, defined as follows:

- *β*_*i*_ is the probability of disease transmission (see Sect. 2.4) in a single contact between a susceptible in age class *i* and a contagious individual (exposed or infected). We assume that exposed individual can transmit the virus at early stages before symptoms develop, as confirmed by epidemiological investigations (e.g., [36]);
- *p*_*i*_ is the susceptibility to infection, taken from [37], Chapter 6, Fig 2;
- *σ* denotes the rate at which individuals move from exposed to infected, while γ is the rate of transition from the compartment of the infected to the compartments of GP/ER treated, ICU treated or recovered. Their values are related to the incubation time *t*_*σ*_ = *σ*^−1^ and the remission time *t*_*γ*_ = *γ*^−1^, assumed to be equal to 1.5 and 4 days respectively [38–40];
- *a*_*i*_ denotes the fraction of infected individuals being treated by general practitioners or in emergency rooms, while *b*_*i*_ is the fraction of infected individuals requiring critical care in ICU. The reference values of these parameters have been taken from [41–44];
- *c*_*i*_ is the vaccination coverage, defined as the fraction of susceptible that get the vaccination. The reference values for the vaccination coverage *c*_ref,*i*_ are taken from [45] (see Sect. 2.3);
- *ϵ*_*i*_(*t*) is the time evolution of the vaccine effectiveness, computed by analysing the results reported in [46] (see Sect. 2.3);
- μ_i_ is the mortality rate, with reference values μ_ref,i_ taken from [47].

**Fig 1:**
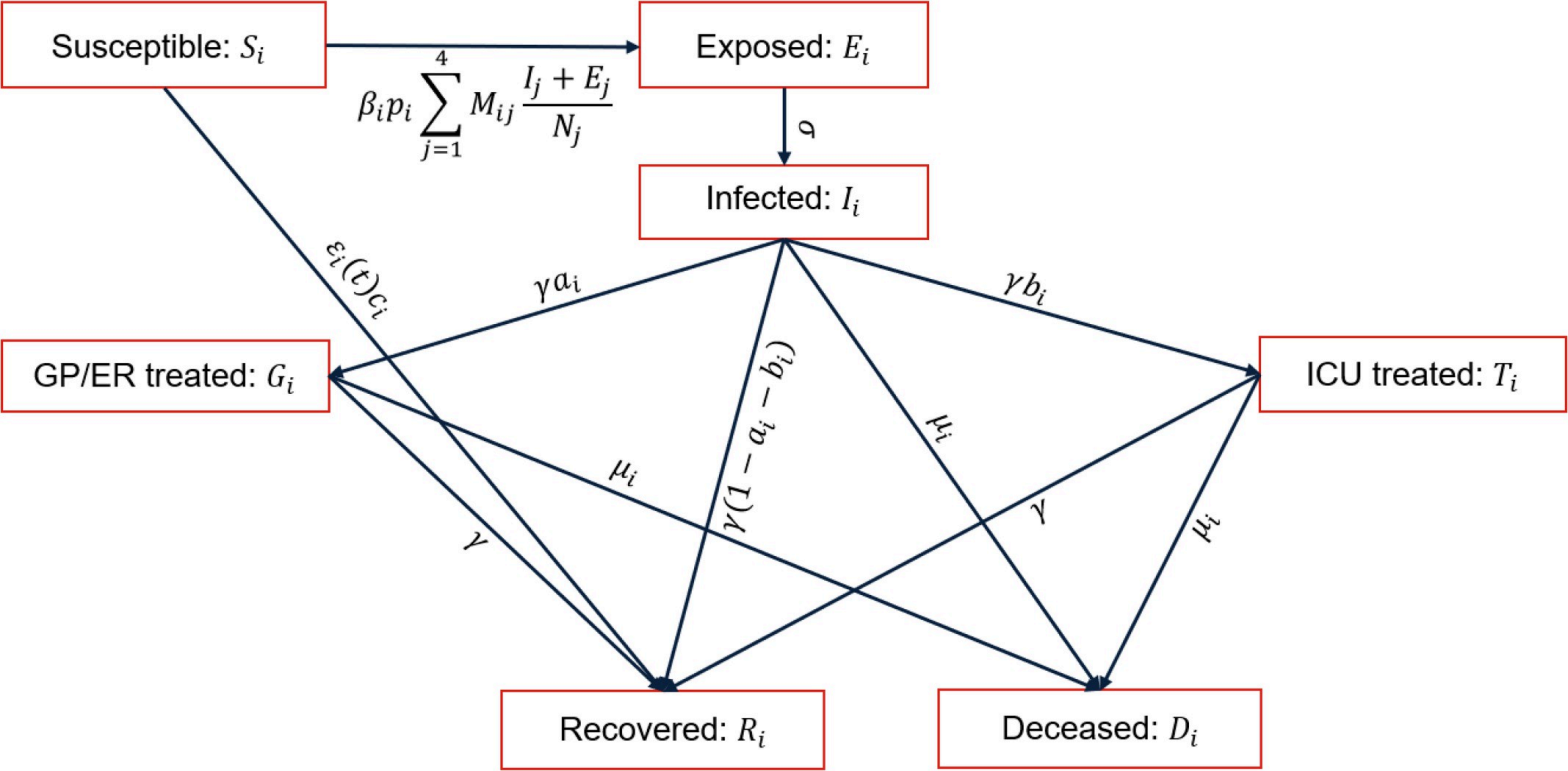
Model scheme. Graphical scheme representing the flow of individuals among compartments.

The reference values of the model parameters are shown in Table 1. A representative stock-flow diagram showing how individuals move through compartments is shown in Fig 1.

**Table 1:** Model parameters.

Age class	Population *N*_*i*_	*C*_ref,*i*_	*a*_*i*_	*b*_*i*_	*p*_*i*_	*μ*_ref,*i*_
0–4 years	2,367,687	0.02657	0.34029	0.18648	1	0.00074
5–14 years	5,594,529	0.02069	0.29472	0.29982	0.45	0.00028
15–64 years	35,564,193	0.05522	0.16019	0.55326	0.3	0.0024
65+ years	16,833,138	0.546	0.18449	0.67425	0.9	0.10814

Reference parameters of the epidemic model for each age class.

The integration of the Eqs 1–7 is carried out by using the standard real-valued variable-coefficient ordinary differential equation solver based on LSODE Fortran library ODEPACK++ [48]. We assume the following initial conditions (i.e., at *t* = 0) for the state variables: *S*_*i*_(0) = *N*_*i*_−1000, *I*_*i*_(0) = 1000, while the rest of state variables are null. We run the simulation of the epidemic over a time period of 100 days, which can be considered a representative period for the duration of seasonal flu epidemics.

### 2.2. Contact matrix

**Fig 2:**
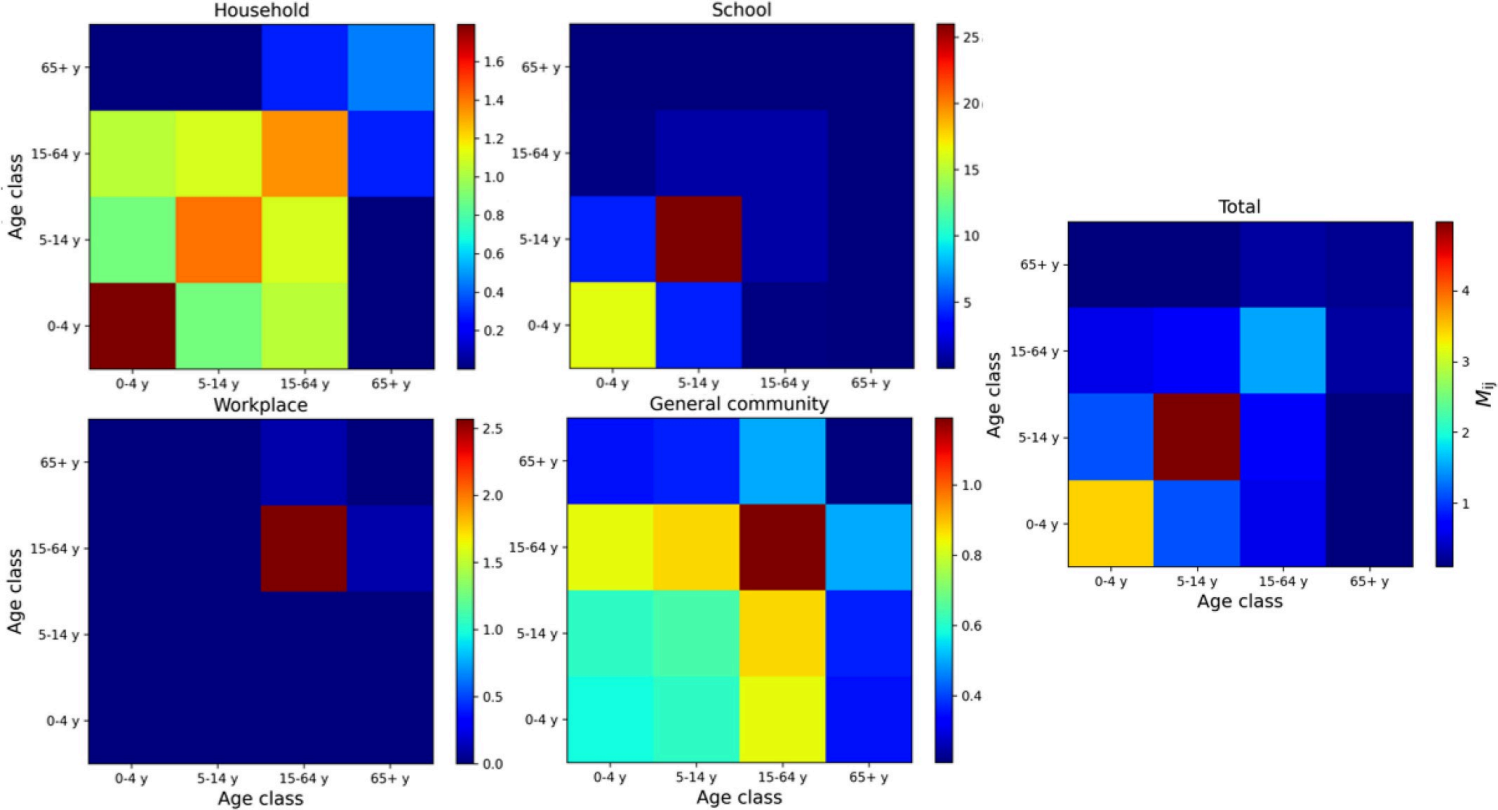
Contact matrices. Contact matrices for the Italian population in different social settings (see the title above each panel). The rightmost panel shows the total contact matrix obtained as a linear combination of the setting-specific matrices [33].

Our model incorporates the impact of social mixing patterns (see Eqs 1 and 2) by means of the contact matrices provided by [33] related to the Italian population. The matrices have been numerically scaled according to the age structure considered in our model. As shown in [33], the contact matrix M_ij_ is computed as a linear combination of four contact matrices related to the expected mixing patterns in different physical environments (household, school, workplace and in the general community), i.e.,

$$M_{ij}=\sum_{K}\delta^{K}\alpha^{K}M_{ij}^{K},$$ (8)

where *α*^K^ are the setting-specified coefficients of the linear combination [33] and *δ*^K^ indicates the corrective factors introduced here to model the adoption of NPIs (see Sect. 2.4). For the purpose of our work, since the impact of the NPIs in the epidemic dynamic are environment specific, we compute the coefficients *α*^K^ of the linear combination by analysing data provided by [33]. The resulting coefficients are *α*^H^ = 0.29, α^S^ = 0.17, *α*^W^ = 0.24 and *α*^G^ = 0.3. These coefficients will be scaled according to the expected impact of the adopted NPI in the mixing patterns, as described in Sect. 2.4. Fig 2 shows the contact matrices related to the different social settings, along with the overall contact matrix adopted in this work.

### 2.3. Vaccination

**Fig 3:**
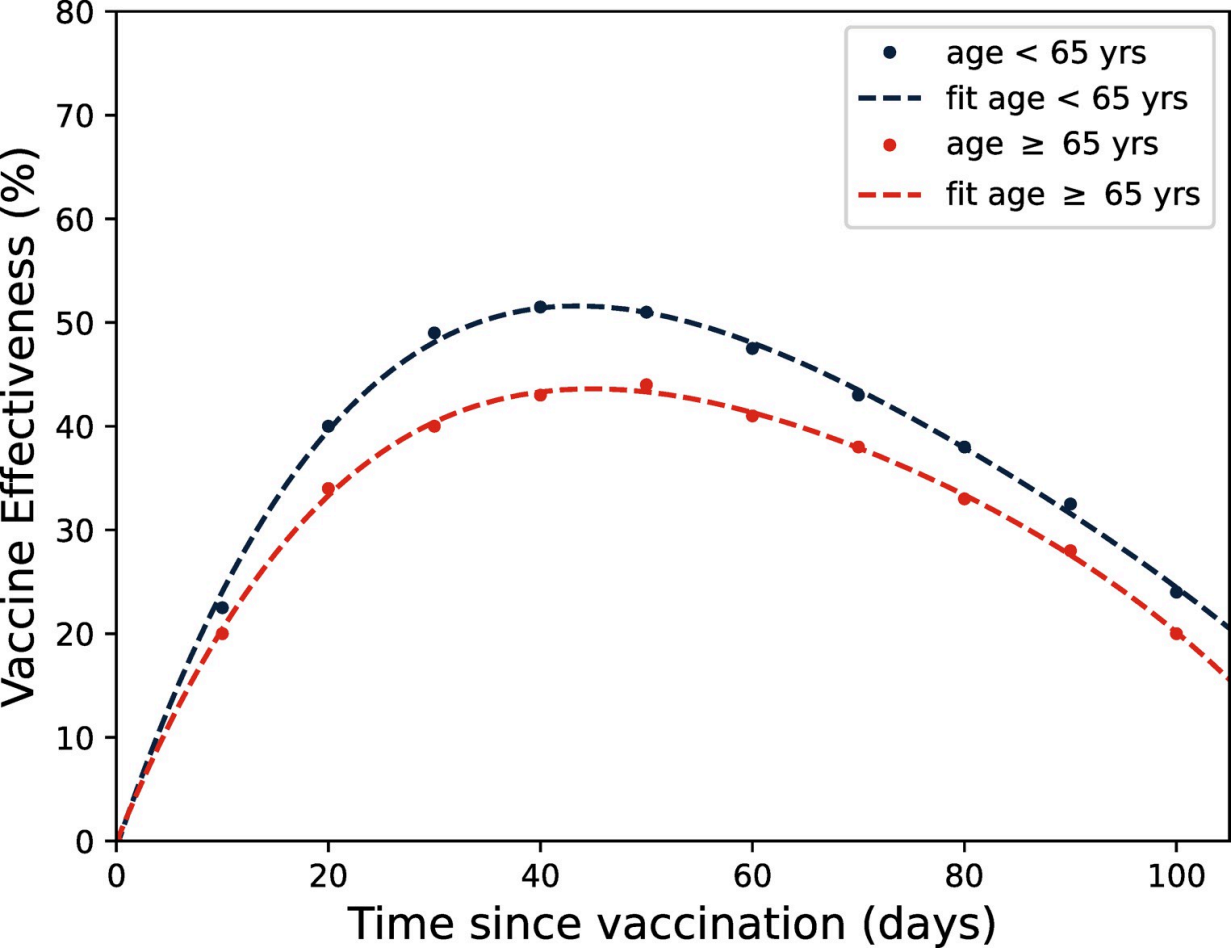
Time evolution of vaccine effectiveness. The dots indicate the time evolution of vaccine effectiveness by time since vaccination among all ages and for individuals aged 65 years and older reported in [46]. Dashed lines denote the least squares fitting functions of the data with fourth order polynomials.

The time evolution of the effectiveness of the influenza vaccination (*ϵ*_*i*_(*t*), see Eqs 1 and 6) is computed by analysing the averaged values of the vaccine effectiveness of the two influenza subtypes H3N2 and H1N1 reported in [46]. In detail, [46] modelled influenza subtype-specific vaccine effectiveness by time since vaccination among all ages and, specifically, for individuals aged 65 years and older (see dotted lines in Fig 3). Due to the sparsity of data provided by [46], we have tested different regression models in order to find the best fitting function by computing the adjusted R-squared metric to measure the goodness of fit. As results, we compute two fourth degree polynomial fitting functions with an adjusted *R*^2^~0.99 (see dashed lines in Fig 3), expressed as follows:

$$\epsilon_{\mathrm{age}<65\mathrm{yrs}}(t)=-1.28\times 10^{-6}\;t^{4}+4.3\times 10^{-4}\;t^{3}-0.056\;t^{2}+3.01\;t-0.64,$$ (9)

$$\epsilon_{\mathrm{age}\geq 65\mathrm{yrs}}(t)=-1.39\times 10^{-6}\;t^{4}+4.05\times \;10^{-4}t^{3}-0.049\;t^{2}+2.51\;t-0.11.$$ (10)

Taking into account the age-structure of the epidemic model presented in this paper, we assume the same vaccine effectiveness time evolution for individuals younger than 65 years.

### 2.4. Model calibration and description of scenarios

The model is calibrated against the age distribution of the cumulative number of ILI cases during the 2018/19 season in Italy, reported in Chapter 6 of [37] (see their Fig 1). The procedure employs a trial-and-error calibration in order to infer the transmission probability β_ref,i_ that reproduces the total number of infected individuals in the different age classes at the end of the epidemic. The resulting model is hereafter referred to as reference model.

As previously mentioned, our analysis aims at exploring a wide range of scenarios in order to infer the impact of control and prevention measures in the evolution of the epidemic, taking into account different seasonal influenza severity. In order to define the potential occurrences of the influenza in terms of seasonal severity, we consider the analysis carried out in [49], who, on the basis of a literature review and of the opinion of a panel of experts, selected the six most likely scenarios that synthesise the possible effects of an influenza outbreak. Out of the six scenarios selected in [49], we assume that the reference model, calibrated on the 2018/19 seasonal influenza, is related to the scenario B shown in [49] (see their Fig 2). In terms of severity, we assume that the reference model is considered as a moderate season. We consider two additional scenarios for the season severity, i.e., mild and severe season, assumed to be related to scenarios A and E in [49], respectively. The severity of the season is modelled by changing the transmission probability *β*_*i*_ and mortality rate *μ*_*i*_ with respect to the reference values *β*_ref,*i*_ and *μ*_ref,*i*_ (see Table 1). Taking into account the clinical attack and case fatality rates of the scenarios presented in [49], we assume that the mild season is characterised by half of the transmission probability and the same mortality rate with respect to the reference scenario, i.e., *β*_mild,*i*_ = 0.5 *β*_*ref*,*i*_ and μ_mild,*i*_ = *μ*_ref,*i*_. The severe season is assumed to be characterised by a transmission probability 50% over the probability of the reference scenario, while the mortality rate is assumed to be three times larger than the reference value, i.e., *β*_severe,*i*_ = 1.5 *β*_ref,*i*_ and *μ*_severe,*i*_ = 3 *μ*_ref,*i*_.

Moreover, our analysis explores a set of scenarios aimed at investigating the impact of social restrictions on population mixing by scaling the setting-specified contact matrices accordingly (see Sect. 2.2). These measures are described as follows:

1. school closures: reduction of contacts at school by 50%, i.e., *δ*^*S*^ = 0.5,
2. partial lockdown: reduction of contacts at school, work and in the general community to 20%, i.e., *δ*^*S*^ = *δ*^*W*^ = *δ*^*G*^ = 0.2.

When not otherwise specified, *δ*^*K*^ are assumed equal to unit values, with *K* = 1,…,4. Furthermore, we explore two additional scenarios to infer the impact of different Vaccination Coverage (VC) with respect to the reference case (*c*_*i*_ = *c*_ref,*i*_, hereafter referred to as standard coverage, see Table 1), i.e.:

1. no VC: *c*_*i*_ = 0 for *i* = 1,…,4;
2. enhanced VC: coverage of 40% for individuals younger than 65 years, *c*_*i*_ = 0.4 for *i* = 1,2,3 and *c*_4_ = *c*_ref,4_ (see Table 1).

Finally, we consider an additional scenario to model the impact of the adoption of PPE and the practice of hand hygiene, which are expected to cover a crucial role in limiting the spread of airborne diseases (e.g., [15]). In this context, [14] observed a significant reduction of 75% in the rate of ILI due to adoption of PPE and the practice of hand hygiene. Therefore, the impact of these measures is modelled by reducing the transmission probability by 75% with respect to the reference case, i.e., *β*_PPE-hygiene,*i*_ = 0.25 *β*_ref,*i*_.

## 3. Results

In this Section we present outcomes of the epidemic dynamical simulations for the set of scenarios described above (see Sect. 2.4). The analysis is aimed at inferring the effectiveness of control and prevention measures in the mitigation of the epidemics. In order to characterize the outcome of the epidemic spread in the different scenarios, we will focus on the time evolution and the cumulative number of the infected and deceased individuals with respect to the number of individuals at the end of epidemics. It is worth remembering that the end of epidemics is assumed to be the 100^th^ day after the beginning of the epidemic. The key advantage of this approach is to analyse the relative trend of the epidemic evolution among different scenarios in order to figure out the impact of the different NPIs and vaccination campaign strategies and compare the effect of each measure in terms of the reduction of the number of infected and deceased individuals.

### 3.1. Impact of vaccination

**Fig 4:**
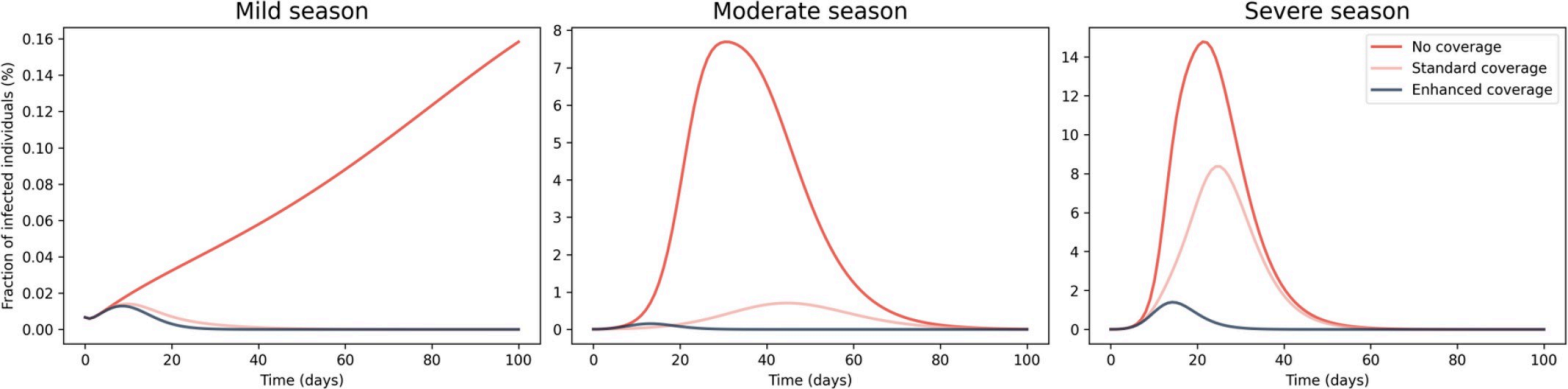
Impact of vaccination on the time evolution of the epidemic. Time evolution of the epidemics for different VC and influenza severity: (left) mild, (center) moderate and (right) severe season. Please note that the scaling of the y-axis is different among each panel and the legend is shown on the right panel.

In Fig 4 is shown the time evolution of the total number of infected individuals with respect to the population number for three different scenarios characterised by different VC, taking into account the three different severity of the season under consideration (see Sect. 2.4). The first days after the outbreak of the epidemic are characterized by an increase of the infected individuals with a rate that increases with the severity of the season and decreases with the enhancement of the vaccination coverage. The time evolution of the fraction of infected individuals peaks at different times and shows a different decrease rate after the peak. In detail, mild seasons (see the left panel of Fig 4) are characterised by a narrow time evolution of the epidemic that vanishes after around 20 days from the beginning of the epidemics, except the case with no VC, where the number of infected individuals requires a longer time to reach the peak with respect to the other scenarios. In the case of moderate and severe seasons (see the central and right panels of Fig 4), the number of infected individuals decreases to null values after about 70–80 days from the beginning of the epidemics. Therefore, the vaccination strictly affects the overall time evolution of the epidemics, leading to a remarkable decrease of both the time peak arrival and the number of infected individuals.

In order to evaluate the overall effect of vaccination, we compute the cumulative fraction of infected and deceased individuals at the end of the epidemic for each age class. Table 2 shows the fraction of infected and deceased individuals with changing VC and season severity for each age class. By averaging the fraction of infected and deceased individuals for each age class over the three season severity scenarios, the absence of vaccination would lead to an increase of the fraction of infected (deceased) individuals by a factor ~1.6 (~1.5) for the age class ≤ 14 years, a factor ~2.4 (~2.2) for those aged 15–64 years and a factor ~6.3 (~5.7) among the elderly with respect to the case of standard VC. The enhancement of the VC leads to a reduction of the fraction of infected (deceased) individuals by a factor ~24.8 (~20.7) for individuals aged less than 5 years, a factor ~35.7 (~36) for those aged 5–14 years, a factor ~23.3 (~22.3) for individuals with age in the range 15–64 years and a factor ~1.6 (~1.7) among the elderly with respect to the case of standard VC.

**Table 2:** Impact of vaccination on the cumulative fraction of infected and deceased individuals.

		Mild season		Moderate season		Severe season	
VC	Age class	I (%)	D (%)	I (%)	D (%)	I (%)	D (%)
No	0–4 years	1.12	0.004	81.2	0.36	95.1	0.64
	5–14 years	0.7	0.001	71.6	0.13	90.7	0.24
	15–64 years	0.62	0.009	51.2	0.83	74.8	1.81
	65+ years	8.02	3.59	99.4	47.9	100	59.8
Standard	0–4 years	0.17	0.0008	30.5	0.14	80.5	0.54
	5–14 years	0.1	0.0001	24	0.045	76.3	0.2
	15–64 years	0.04	0.0006	7.5	0.12	44.3	1.07
	65+ years	0.16	0.08	3	1.69	29.4	17.6
Enhanced	0–4 years	0.09	0.0004	0.47	0.002	3.92	0.02
	5–14 years	0.05	0.00008	0.27	0.0005	2.49	0.006
	15–64 years	0.02	0.0003	0.2	0.003	2	0.05
	65+ years	0.14	0.07	2	0.99	17.4	10.4

Cumulative fraction of infected (I) and deceased (D) individuals among all age classes for different season severity and vaccination coverage.

As expected, the impact of standard vaccination is stronger for elderly people (age ≥ 65 years), reaching an average reduction over the three scenario of season severity of ~88% of the infected and deceased population with respect to the scenario without vaccination, while the reduction of infected and deceased non-elderly subjects reaches values of ~73% for those aged 15–64 years and of ~55% for infants and children. This is due to the relatively larger vaccination coverage for elderly subjects (see Table 1). Moreover, the enhancement of the VC leads to a further reduction of the fraction of infected and deceases population among all age classes in the range between ~92% and ~97% with respect to the case without vaccination.

By computing the cumulative fraction over all age classes, mild season epidemic evolution with standard VC shows similar results among all age classes compared to the one with enhanced coverage, with a cumulative fraction of infected people of around ~0.08% and ~0.06% with standard and enhanced coverage, respectively (see pink and blue lines in the left panel of Fig 4). For moderate seasons, the standard coverage leads to an infection reduction of ~87%, thus decreasing the cumulative fraction of infected individuals from ~67% (in the case of no coverage) to ~8.6% with respect to the total population. By enhancing the coverage, the infected fraction decreases up to ~0.72%.

During severe season, the standard coverage produces a reduction of infection by ~47%. In detail, the cumulative fraction of infected people decreases from ~84% (in the case of no coverage) to ~44% of the total population, while the enhanced coverage produces a further reduction up to ~6.4%.

### 3.2. Impact of NPIs

**Fig 5:**
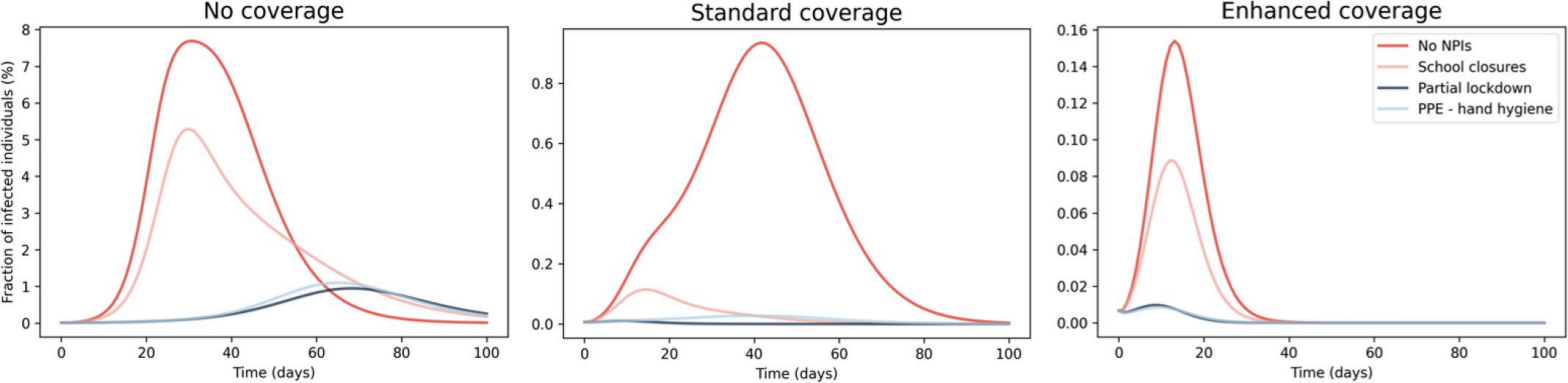
Impact of NPIs on the time evolution of a moderate influenza season for different VC. Time evolution of the epidemics during a moderate season including the effects of different NPIs (lines in each panel) and vaccination with (left) no, (center) standard and (right) enhanced coverage. Please note that the scaling of the y-axis is different among each panel and the legend is shown on the right panel.

In this Section we compare the impact of the adoption of single NPIs in the mitigation of the spread of disease with respect to the vaccination in moderate and severe seasons, taking into account, additionally, the combined effect of NPIs and vaccination. Fig 5 shows the time evolution of the total fraction of infected individuals during a moderate influenza season for different VC and adopting different NPIs. By comparing the different evolution of the epidemic due to the adoption of single NPI (lines in each panel of Fig 5), it can be noticed that the peak of the infection is reached at different time after the beginning of the epidemics and the overall duration of the spread of the disease changes among the different scenarios. A standard VC leads to a reduction of the peak of the infection towards a value similar to the peak of the infection in the case of no coverage with the adoption of lockdown and PPE—hand hygiene (compare blue and light blue lines in the left panel with the red line in the central panel of Fig 5).

Moreover, the impact of the enhancement of the VC is similar to the effect produced by the standard vaccination and school closures (compare the light red line in the central panel with the red line in the right panel of Fig 5).

Table 3 shows the age-structured fraction of infected and deceased individuals with different NPIs and VC during a moderate influenza season.

**Table 3:** Impact of the NPIs for different VC on the cumulative fraction of infected and deceased individuals during a moderate influenza season.

		No NPIs		School closure		Partial lockdown		PPE—hand hygiene	
VC	Age class	I (%)	D (%)	I (%)	D (%)	I (%)	D (%)	I (%)	D (%)
No	0–4 years	81.2	0.36	46.6	0.2	1.4	0.006	59	0.26
	5–14 years	71.6	0.13	33.6	0.06	0.84	0.001	6.3	0.01
	15–64 years	51.2	0.83	34.6	0.56	2.22	0.03	4.5	0.07
	65+ years	99.4	47.9	97.4	47	45.4	21.4	29.6	14.05
Standard	0–4 years	30.5	0.14	1.1	0.005	0.08	0.004	4.77	0.02
	5–14 years	24	0.045	0.7	0.001	0.04	0.00007	0.36	0.0006
	15–64 years	7.5	0.12	0.5	0.008	0.02	0.0004	0.14	0.002
	65+ years	3	1.69	1.5	0.72	0.12	0.05	0.12	0.05
Enhanced	0–4 years	0.47	0.002	0.17	0.0007	0.07	0.0003	0.26	0.001
	5–14 years	0.27	0.0005	0.09	0.0002	0.03	0.00005	0.03	0.00006
	15–64 years	0.2	0.003	0.1	0.002	0.01	0.0002	0.01	0.0002
	65+ years	2	0.99	1.2	0.59	0.11	0.05	0.07	0.04

Cumulative fraction of infected (I) and deceased (D) individuals for moderate influenza season scenarios where different NPIs and VC are adopted.

**Fig 6:**
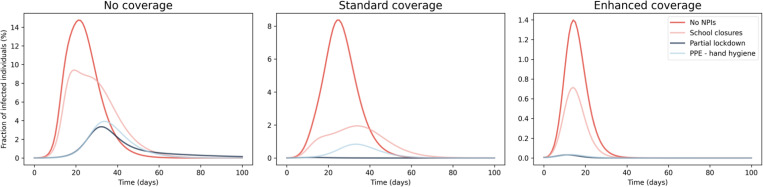
Impact of NPIs on the time evolution of a severe influenza season for different VC. Time evolution of the epidemics during a severe season including the effects of different NPIs (lines in each panel) and vaccination with (left) no, (center) standard and (right) enhanced coverage. Please note that the scaling of the y-axis is different among each panel and the legend is shown on the right panel.

In Fig 6 is shown the time evolution of the total fraction of infected individuals during a severe influenza season for different VC and adopting different NPIs. Similarly, to the case of moderate influenza season shown in Fig 6, the peak of infection is reached at different time after the beginning of the epidemics and the overall duration of the spread of the disease changes among the different scenarios. In the case of no coverage (see left panel of Fig 6), the adoption of PPE, hand hygiene and partial lockdown leads to a larger reduction of infection peak with respect to the one produced by standard VC (see the left and central panel of Fig 6), contrary to what observed for moderate influenza season. Moreover, the impact of the enhancement of the VC is similar to the effect produced by the combination of standard vaccination and the adoption of PPE and hand hygiene (compare the light blue line in the central panel with the red line in the right panel of Fig 6). Moreover, in case of no and standard coverage, Figs 5 and 6 shows that NPIs are useful in delaying the epidemic peak, while in case of enhanced VC the adoption of NPIs do not affect the timing of epidemics. Table 4 shows the amount of the overall reduction of the spread of epidemics with different NPIs and VC for different age classes during a severe influenza season.

**Table 4:** Impact of the NPIs for different vaccination coverage during a severe influenza season.

		No NPIs		School closure		Partial lockdown		PPE—hand hygiene	
VC	Age class	I (%)	D (%)	I (%)	D (%)	I (%)	D (%)	I (%)	D (%)
No	0–4 years	95.1	0.64	81.6	0.55	13.6	0.09	87.7	0.6
	5–14 years	90.7	0.24	69.5	0.18	8.01	0.02	17.3	0.05
	15–64 years	74.8	1.81	67.6	1.63	11.05	0.26	11.8	0.28
	65+ years	100	59.8	100	59.8	82.5	49.3	62.1	37.2
Standard	0–4 years	80.5	0.54	39.1	0.26	0.22	0.003	58.4	0.4
	5–14 years	76.3	0.2	30.7	0.08	0.11	0.0002	7.76	0.02
	15–64 years	44.3	1.07	18.2	0.44	0.1	0.002	3.34	0.08
	65+ years	29.4	17.6	14.9	8.91	0.51	0.3	1.26	0.75
Enhanced	0–4 years	3.92	0.03	0.91	0.006	0.1	0.0007	1.1	0.007
	5–14 years	2.49	0.006	0.55	0.001	0.05	0.0001	0.09	0.0002
	15–64 years	2	0.05	0.86	0.02	0.04	0.001	0.05	0.001
	65+ years	17.4	10.4	10.1	6	0.5	0.28	0.31	0.18

Cumulative fraction of infected (I) and deceased (D) individuals for severe influenza season scenarios where different NPIs and VC are adopted.

## 4. Discussion

By averaging the results related to the three cases of VC for moderate (severe) influenza seasons, the impact of school closure leads to a reduction of the fraction of infected and deceased population by a factor ~2.3 (~1.5) for individuals aged less than 5 years, a factor ~2.8 (~1.7) for those aged 5–14 years, a factor ~ 1.7 (~1.4) for individuals with age in the range 15–64 years and any reduction among the elderly with respect to the scenario without the adoption of any NPIs.

Moreover, partial lockdown mitigates the spread of moderate (severe) seasonal influenza epidemics by reducing the fraction of infected and deceased individuals by a factor ~73 (~12) for individuals aged less than 5 years, a factor ~107 (~23) for those aged 5–14 years, a factor ~28 (~10) for individuals with age in the range 15–64 years and a factor ~2.3 (~1.7) among the elderly with respect to the scenario without the adoption of any NPIs.

Finally, the adoption of PPE and the practice of hand hygiene during a moderate (severe) influenza season leads to a reduction of infected and deceased individuals by a factor ~1.7 (~1.2) for individuals aged less than 4 years, a factor ~14 (~7) for those aged 5–14 years, a factor ~13 (~8) for individuals with age in the range 15–64 years, and a factor ~3.5 (~2.3) among the elderly with respect to the scenario without the adoption of any NPIs. Therefore, by considering each age class, partial lockdown is the most effective measure in containing the epidemic, especially for individuals aged less than 64 years. By contrast, PPE and hand practice hygiene are the most effective NPIs for elderly individuals for all the cases of season severity.

By considering the total population regardless of age classes, our results show that the adoption of PPE and the practice of hand hygiene are the most effective NPIs in terms of reduction of infection and death during moderate influenza seasons in absence of vaccination, producing a reduction of infected (deceased) individuals by ~79% (~71%) with respect to the scenario without the adoption of NPIs and vaccination. By contrast, school closures lead to a reduction of ~22% (~3.5%) of infected (deceased) cases.

However, as mentioned in Sect. 3.1, the impact of vaccination with a standard coverage leads to a reduction of ~87% (~95%) of the total fraction of infected (deceased) individuals with respect to the scenario without any vaccination and NPI. The reduction of the spread of the disease is therefore larger in the case a standard vaccination coverage is achieved with respect to the scenario where vaccination is not undertaken and with the adoption of NPIs, regardless of the nature of the NPI. An enhancement of the vaccination coverage further reduces the spread of the disease by limiting the cases of infected (deceased) individuals up to ~98% (~97%) with respect to the number of infected individuals in cases where vaccination campaigns are not undertaken. Therefore, in case of moderate influenza season, vaccination campaigns with, at least, a standard coverage make the adoption of NPIs unnecessary.

However, in both cases of influenza severity, a further reduction of infected (deceased) individuals up to 99% (~100%) is obtained when standard vaccination is combined with the adoption of NPIs, regardless of which kind of NPI is adopted.

In the case of severe influenza seasons and no vaccination, PPE and the practice of hand hygiene are still the most effective NPIs in terms of reduction of the total fraction of infected and deceased individuals, similarly to the case of moderate influenza season. In detail, the adoption of PPE and the practice of hand hygiene produce a reduction of infected (deceased) individuals by ~65% (~41%), while partial lockdown leads to a reduction up to ~63% (~21%) with respect to the scenario without the adoption of NPIs. These results confirm that PPE and hand hygiene are valuable measures to reduce the spread of the disease without requiring any mass intervention such as lockdown.

As mentioned in Sect. 3.1 a standard vaccination coverage in severe influenza season leads to a reduction of infected (deceased) individuals by ~47% (~69%), while the enhanced vaccination produces a decrease of infected (deceased) individuals by ~92% (~84%). Therefore, in case of severe influenza season, the enhancement of the vaccination coverage is necessary to avoid the adoption of NPIs. Finally, by combining standard (enhanced) vaccination with the adoption of NPIs, we observe a further reduction of epidemic in the range between ~ 77% (~95%) and ~ 99% (~99%) for infected individuals and in the range between ~ 85% (~90%) and ~100% (~100%) for deceased individuals, regardless of the nature of the adopted NPI.

## 5. Conclusion

The main aim of this paper is to attempt to answer the question of how currently adopted public health strategies are effective against the variety of expected scenarios of influenza in the upcoming season 2021/22. The exploration of these scenarios is needed in order to evaluate a more mindful adoption of NPIs and, possibly, a revision of currently adopted vaccination campaigns in order to prevent the spread of the epidemic by implementing appropriate vaccination campaigns and social interventions.

To this aim, we have developed an age-structured SEIR model that includes the modelling of prevention and control measures, as well as the effect of social mixing patterns and the time evolution ofvaccine effectiveness in the epidemic dynamics. We present a thorough incidence analysis for three different scenarios of seasonal influenza severity under a number of hypothetical government intervention strategies (i.e. vaccination and NPIs). Based on the results of this study, vaccination campaigns with a standard coverage would produce a remarkable impact on the spread of the disease in the case of a moderate influenza season, making the adoption of NPIs unnecessary. On the contrary, NPIs are necessary in case of severe seasonal epidemics, where a standard vaccination coverage would not be sufficiently effective in fighting the disease. In this case, the combination of a vaccination with standard coverage and NPIs would lead to a significant containment of the disease. However, our results show that the enhancement of the vaccination coverage would reduce the need to adopt NPIs, thus limiting the economic and social impacts that NPIs might produce. Encompassing all the epidemic outcomes explored in this paper, it can be concluded that while the explored NPIs are effective in reducing the transmissions and mitigating the healthcare burden, it is clear that a vaccination campaign with a proper coverage is the only way to contain the epidemic.

This approach suffers from some uncertainties that might affect our result. Firstly, the model assumes a population with no migration, births or deaths from causes other than the epidemic and assumes immunity to the disease upon recovery. Concerning the social mixing, the model is based on the setting-specific social interaction matrices provided by [33] which are related to four social settings (see Sect. 2.2. The adoption of NPIs that limit the social mixing due to public mobility restrictions (i.e., school closures and partial lockdown) are modelled through a rescaling approach (see Sect. 2.4).

However, this approach might be simplistic since it does not take into account the adherence of individuals to such restrictions. By accessing data related to contact tracing for confirmed cases and by carrying out large scale public surveys, the social mixing model adopted in this paper can be improved. Moreover, the model does not consider the stochastic factors, which can strongly influence the dynamics of the epidemic in the early and final stages, when there are a few numbers of infected individuals [23].
